# Supplementary material for: Individual recognition in a jumping spider (Phidippus regius)
Source: eLife. 2025 Nov 11;13:RP97146. doi: 10.7554/eLife.97146 (PMC12604854; doi:10.7554/eLife.97146)
Supplement: MDAR checklist [file elife-97146-mdarchecklist1.pdf]

Materials Design Analysis Reporting (MDAR) Checklist for Authors

The MDAR framework establishes a minimum set of requirements in transparent reporting mainly applicable to studies in the life sciences.

eLife asks authors to **provide detailed information within their article** to facilitate the interpretation and replication of their work. Authors can also upload supporting materials to comply with relevant reporting guidelines for health-related research (see EQUATOR Network), life science research (see the BioSharing Information Resource), or animal research (see the ARRIVE Guidelines and the STRANGE Framework; for details, see *eLife*'s Journal Policies). Where applicable, authors should refer to any relevant reporting standards materials in this form.

For all that apply, please note **where in the article** the information is provided. Please note that we also collect information about data availability and ethics in the submission form.

Materials:

**Newly created materials**	**Indicate where provided: section/figure legend**	**N/A**
The manuscript includes a dedicated "materials availability statement" providing transparent disclosure about availability of newly created materials including details on how materials can be accessed and describing any restrictions on access.	Section: Additional information: "Codes and materials are available (https://osf.io/gpnct/)"<br>Main text: "Supplementary Videos in the OSF repository"	

**Antibodies**	**Indicate where provided: section/figure legend**	**N/A**
For commercial reagents, provide supplier name, catalogue number and RRID, if available.		**N/A**

**DNA and RNA sequences**	**Indicate where provided: section/figure legend**	**N/A**
Short novel DNA or RNA including primers, probes: Sequences should be included or deposited in a public repository.		**N/A**

**Cell materials**	**Indicate where provided: section/figure legend**	**N/A**
Cell lines: Provide species information, strain. Provide accession number in repository OR supplier name, catalog number, clone number, OR RRID.		**N/A**
Primary cultures: Provide species, strain, sex of origin, genetic modification status.		**N/A**

Experimental animals	Indicate where provided: section/figure legend	N/A
Laboratory animals or Model organisms: Provide species, strain, sex, age, genetic modification status. Provide accession number in repository OR supplier name, catalog number, clone number, OR RRID.	Materials and methods → Subjects:<br>Species: *Phidippus regius* (jumping spider)<br>Strain: N/A (no strain designation)<br>Sex: Both sexes, assigned to sex-specific groups ("three groups female, two male" in Experiment 1; "two groups per sex" in Experiment 2).<br>Age: Adults<br>Genetic modification status: None (wild type, no modifications)<br>Accession number / supplier / catalog number / clone / RRID: N/A (laboratory-bred animals, not purchased from a commercial supplier or maintained as a formal strain)	
Animal observed in or captured from the field: Provide species, sex, and age where possible.		**N/A**

Plants and microbes	Indicate where provided: section/figure legend	N/A
Plants: provide species and strain, ecotype and cultivar where relevant, unique accession number if available, and source (including location for collected wild specimens).		**N/A**
Microbes: provide species and strain, unique accession number if available, and source.		**N/A**

Human research participants	Indicate where provided: section/figure legend) or state if these demographics were not collected	N/A
If collected and within the bounds of privacy constraints report on age, sex, gender and ethnicity for all study participants.		**N/A**

Design:

Study protocol	Indicate where provided: section/figure legend	N/A
If the study protocol has been pre-registered, provide DOI. For clinical trials, provide the trial registration number OR cite DOI.		**N/A**

**Laboratory protocol**	**Indicate where provided: section/figure legend**	**N/A**
Provide DOI OR other citation details if detailed step-by-step protocols are available.		**N/A**

**Experimental study design (statistics details) ***		
**For in vivo studies: State whether and how the following have been done**	**Indicate where provided: section/figure legend. If it could have been done, but was not, write “not done”**	**N/A**
Sample size determination	Section: Materials and methods → Data logging and analysis:<br><br>“No statistical methods were used to predetermine sample size.”<br><br>Sample sizes were chosen in line with prior studies on related topics in spiders, which typically employ N≈20-40 per experiment. We ensured sufficient individuals to replicate results across two independent experiments.	
Randomisation	Section: Materials and methods → Data logging and analysis:<br>“The experiments were not randomized.”<br><br>Randomisation was not applied because stimuli and trial orders were fixed across individuals, and outcomes (approach/avoidance behaviour) were objectively coded.	
Blinding	Section: Materials and methods → Data logging and analysis: The investigators were not blinded to allocation during experiments and outcome assessment.<br><br>Blinding was not applied because stimuli and trial orders were fixed across individuals, and outcomes (approach/avoidance behaviour) were objectively coded.	
Inclusion/exclusion criteria	Section: Materials and methods → Data logging and analysis: There is no explicit mention of inclusion or exclusion criteria for animals, trials, or data points.<br>The text, however, specifies:<br>Subjects: 36 adult, laboratory-bred *Phidippus regius* spiders.<br>Analyses: all trials were included as per the defined habituation-dishabituation design.	

**Sample definition and in-laboratory replication**	**Indicate where provided: section/figure legend**	**N/A**
State number of times the experiment was replicated in the laboratory.	Experiment 1: “In the first experiment, we divided a total of 20 individuals into five groups of four individuals each. Each individual of each group was exposed … resulting in six trials per session, equivalent to one hour of recording. We repeated this procedure twice, resulting in 18 trials across three sessions and an exact repetition of a given trial (and pairing of individuals) in 1-hour intervals.”<br>Experiment 2: “We re-ran the experiment in an additional 16 spiders, arranged to four groups … and added a memory dishabituation [long-term] trial at the end of Session 3.”<br>“Two amendments were made in Experiment 2: (a) we ran two groups of four individuals in parallel, and (b) we introduced additional cross-group trials … at the end of Session 3.”<br><br>Experiment 1: Each trial sequence repeated across three sessions per group (total of 18 trials per individual).<br><br>Experiment 2: Replicated with 16 new spiders, again three sessions per group plus additional cross-group trials.<br><br>Overall: The study protocol was replicated independently in two experiments (Experiment 1 and Experiment 2), each running three sessions.	
Define whether data describe technical or biological replicates.	Material and methods: “Subjects: 36 *Phidippus regius* spiders (biological individuals)”<br><br>All replicates were biological (different spiders). No technical replicates were performed.	

**Ethics**	**Indicate where provided: section/submission form**	**N/A**
Studies involving human participants: State details of authority granting ethics approval (IRB or equivalent committee(s), provide reference number for approval.		**N/A**
Studies involving experimental animals: State details of authority granting ethics approval (IRB or equivalent committee(s), provide reference number for approval.	Section: Ethical approval: “According to Taiwan’s Animal Protection Act, issued by the Council of Agriculture (Executive Yuan), experiments on invertebrates are allowed to be conducted without any special permission in Taiwan.”	

Studies involving specimen and field samples: State if relevant permits obtained, provide details of authority approving study; if none were required, explain why.		**N/A**

**Dual Use Research of Concern (DURC)**	**Indicate where provided: section/submission form**	**N/A**
If study is subject to dual use research of concern regulations, state the authority granting approval and reference number for the regulatory approval.		**N/A**

Analysis:

**Attrition**	**Indicate where provided: section/figure legend**	**N/A**
Describe whether exclusion criteria were pre-established. Report if sample or data points were omitted from analysis. If yes, report if this was due to attrition or intentional exclusion and provide justification.	Not explicitly stated in the manuscript: No exclusion criteria were pre-established, and no animals or data points were omitted from the analyses.	

**Statistics**	**Indicate where provided: section/figure legend**	**N/A**
Describe statistical tests used and justify choice of tests.	Materials and methods → Data logging and analysis: "…We used linear mixed-effects models, where the differences in proportions served as the dependent variable. We fitted two separate models… fixed effects: Distance, Session, Condition; two-way and three-way interactions… Sex and Subject as random factors. We created a null model… Using likelihood ratio test (LRT), we compared the null models with the corresponding full models… An additional analysis of variance (ANOVA) was performed comparing the dishabituation [long-term] trials at the end of Session 3 with the dishabituation [short-term] trials from Session 3 as a function of distance."<br><br>Justification: We used linear mixed-effects models (LME) because they appropriately handle repeated measures within subjects and allow random effects for subject identity and sex. Full vs null models were compared using likelihood ratio tests (LRTs) to assess whether including condition and session significantly improved model fit. An additional ANOVA was used to test the specific planned comparison between long-term and short-term dishabituation trials within Session 3.	

	Experiment 1 (N = 20 spiders, 3 sessions):<br>Linear mixed-effects model revealed a significant interaction between distance × condition (habituation vs. dishabituation):<br><br>Likelihood ratio test (LRT): $\chi^2(3) = 63.66$, $p < 0.001$<br>A significant distance × condition × session interaction indicated the effect varied across sessions:<br><br>LRT: $\chi^2(6) = 34.14$, $p < 0.001$<br><br>***Experiment 2*** (N = 16 spiders, 3 sessions + long-term dishabituation):<br>Replicated the distance × condition effect:<br><br>LRT: $\chi^2(3) = 29.52$, $p < 0.001$<br><br>Long-term dishabituation (novel conspecific at end of Session 3) vs. short-term dishabituation (same session) showed a stronger rebound:<br><br>ANOVA: $F(3,127) = 3.91$, sum sq = 0.92, mean sq = 0.31, $p < 0.01$<br><br>***Data presentation:***<br>Effects are visualised in Figure 2 as mean ± SEM of proportional distances (binned into four distance categories)	

**Data availability**	**Indicate where provided: section/submission form**	**N/A**
For newly created and reused datasets, the manuscript includes a data availability statement that provides details for access (or notes restrictions on access).	Section: Additional information: “Codes and materials are available (https://osf.io/gpnct/)”<br>Main text: “Supplementary Videos in the OSF repository”<br>Restrictions: Access is open.<br>Reused data: Not applicable: all data are newly created in this study.	
When newly created datasets are publicly available, provide accession number in repository OR DOI and licensing details where available.	https://osf.io/gpnct/	
If reused data is publicly available provide accession number in repository OR DOI, OR URL, OR citation.		**N/A**

**Code availability**	**Indicate where provided: section/figure legend**	**N/A**

For any computer code/software/mathematical algorithms essential for replicating the main findings of the study, whether newly generated or re-used, the manuscript includes a data availability statement that provides details for access or notes restrictions.	Section: Additional information: "Codes and materials are available (https://osf.io/gpnct/)." Identifier: DOI 10.17605/OSF.IO/GPNCT Licensing details (e.g., CC BY 4.0 for data/code, or a specific OSF license).	
Where newly generated code is publicly available, provide accession number in repository, OR DOI OR URL and licensing details where available. State any restrictions on code availability or accessibility.	Section: Additional information: "Codes and materials are available (https://osf.io/gpnct/)." Identifier: DOI 10.17605/OSF.IO/GPNCT Licensing details (e.g., CC BY 4.0 for data/code, or a specific OSF license). Restrictions: no restrictions	
If reused code is publicly available provide accession number in repository OR DOI OR URL, OR citation.		**N/A**

Reporting:

The MDAR framework recommends adoption of discipline-specific guidelines, established and endorsed through community initiatives.

**Adherence to community standards**	**Indicate where provided: section/figure legend**	**N/A**
State if relevant guidelines (e.g., ICMJE, MIBBI, ARRIVE, STRANGE) have been followed, and whether a checklist (e.g., CONSORT, PRISMA, ARRIVE) is provided with the manuscript.	Ethical approval section:<br><br>"The study adhered to the ARRIVE and STRANGE guidelines for reporting animal research. No formal reporting checklist is included with the manuscript."	

---

* We provide the following guidance regarding transparent reporting and statistics; we also refer authors to Ten common statistical mistakes to watch out for when writing or reviewing a manuscript.

Sample-size estimation

- You should state whether an appropriate sample size was computed when the study was being designed
- You should state the statistical method of sample size computation and any required assumptions
- If no explicit power analysis was used, you should describe how you decided what sample (replicate) size (number) to use

Replicates

- You should report how often each experiment was performed
- You should include a definition of biological versus technical replication
- The data obtained should be provided and sufficient information should be provided to indicate the number of independent biological and/or technical replicates
- If you encountered any outliers, you should describe how these were handled
- Criteria for exclusion/inclusion of data should be clearly stated
- High-throughput sequence data should be uploaded before submission, with a private link for reviewers provided (these are available from both GEO and ArrayExpress)

Statistical reporting

- Statistical analysis methods should be described and justified
- Raw data should be presented in figures whenever informative to do so (typically when N per group is less than 10)
- For each experiment, you should identify the statistical tests used, exact values of N, definitions of center, methods of multiple test correction, and dispersion and precision measures (e.g., mean, median, SD, SEM, confidence intervals; and, for the major substantive results, a measure of effect size (e.g., Pearson's r, Cohen's d)
- Report exact p-values wherever possible alongside the summary statistics and 95% confidence intervals. These should be reported for all key questions and not only when the p-value is less than 0.05.

Group allocation

- Indicate how samples were allocated into experimental groups (in the case of clinical studies, please specify allocation to treatment method); if randomization was used, please also state if restricted randomization was applied
- Indicate if masking was used during group allocation, data collection and/or data analysis
